# 

**Published:** 1896-09-26

**Authors:** 


**Notices of Births, Deaths, and Marriages** are inserted in this column at a charge of 2s. 6d. for an announcement not exceeding four lines.

### NOTICE TO CORRESPONDENTS.

All MS., Letters, Books for Review, and other matters intended for the Editor should be addressed The Editor, The Lodge, Porchester Square, London, W.

The Editor cannot undertake to return rejected MS., even when accompanied by stamped directed envelope.

**Notice to Advertisers and Subscribers.**

All Advertisements, Orders for Copies of The Hospital and Business Communications should be addressed to **THE MANAGER, (Not to The Editor),** The Hospital, 428, Strand, London, W.C.

The Cost of Subscription, post free, is as follows:—Per week, 2½d.; per quarter, 2s. 8d.; per half-year, 5s. 3d.; per annum, 10s. 6d. Foreign:—Per Annum, 15s. 2d.; payable, in all cases, in advance.

**IMPORTANT NOTICE.**

On and after October 1st, 1896, the EDITORIAL and PUBLISHING OFFICES of "THE HOSPITAL" will be TRANSFERRED to their new premises "THE HOSPITAL" BUILDING, 28 and 29, SOUTHAMPTON STREET, STRAND, LONDON, W.C.

**NOTICE.—Vol. XX. of "The Hospital" (April to September, 1896), in handsome cloth binding, will be on sale at the Office of this Journal, early in October, Price 7s. 6d. Cases for binding the Numbers contained in this Volume, 1s. 6d. Index to Vol. XX., 2½d., post free.**

**The Monthly Part for October will be ready October 1st. Price 6d., by post 8d.**

### THE STUDENT OF MEDICINE.

### APPOINTMENTS.

H. B. Angus, M.B., B.S.Dunelm, M.R.C.S., L.R.C.P., appointed Honorary Assistant Surgeon to the Royal Infirmary, Newcastle-upon-Tyne. H. H. Back, M.B.Lond., M.R.C.S.Eng., appointed Medical Officer of Health to the Aylsham District Council. C. E. Bashall, M.R.C.S., L.R.C.P.Lond., appointed Medical Officer for the Workhouse of the Kingsbridge Union. Dr. H. Bryant, appointed Medical Officer for the Fontmell District of the Shaftesbury Union. C. Caldecott, M.B., B.S.Lond., M.R.C.S., L.S.A., appointed Resident Medical Superintendent of the Earlswood Asylum for Idiots and Imbeciles, Redhill, Surrey. Dr. Cartwright, appointed Medical Officer of Health to the Wig-

**THE STUDENT OF MEDICINE**—*continued.*

more Rural District. T. M. Clayton, M.B., B.S.Durh., appointed Medical Officer of Health to the Felling Urban District Council. W. H. Coates, M.A., M.B., M.R.C.S., L.R.C.P., L.S.A., L.S.Sc., appointed Acting Medical Officer of Health and Medical Officer to Patrington Union, and also appointed by the Elder Brethren of Trinity House to be Surgeon in charge of Spurn Lighthouse. W. C. G. Collins, L.R.C.P.Edin., M.R.C.S. Eng., appointed Medical Officer for the Aylesford District of the Malling Union. C. E. Fish, B.A.Camb., M.B., B.C., appointed Assistant Medical Officer of the Workhouse and Assistant to the Superintendent of the Infirmary of the Paddington Parish. J. Glaister, M.D.Aberd., appointed Medical Officer of Health for Clerkenwell. E. S. Hemsted, M.R.C.S.Eng., L.R.C.P.Lond., appointed Medical Officer for the Kintbury District of the Hungerford Union. H. Jackman, L.R.C.P., L.R.C.S.Edin., appointed Medical Officer for the Tenth District of the Hackney Union. E. W. James, M.R.C.S.Eng., appointed Medical Officer for the Hardingham District of the Mitford and Launditch Union. Mr. Johnstone, appointed Medical Officer for the Horsmonden District of the Tonbridge Union. T. C. Lawson, M.R.C.S.Eng., appointed Medical Officer for the Bradfield District of the Hardingstone Union. W. H. Macpherson, M.R.C.S., L.R.C.P.Lond., appointed Medical Officer for the Grays District of the Henley Union. F. W. Mais, L.R.C.P., L.R.C.S.Edin., appointed Medical Officer for the Thorner District of the Wetherby Union. Dr. C. A. Morgan, appointed Medical Officer for the Thorncombe District of the Beaminster Union. W. Murray, M.A., M.B. and C.M.Aberd., appointed Assistant House-Surgeon to the Preston and County of Lancaster Royal Infirmary. J. M. Rogers-Tillstone, M.R.C.S., L.R.C.P.Lond., appointed Medical Officer for the Second East Malling District of the Malling Union. J. L. Rubel, M.R.C.S., L.R.C.P.Lond., appointed Medical Officer to the Second Division of the Hougham District of the Dover Union. W. W. Shackleton, M.D.Dub., appointed Medical Officer of the Schools of the St. Pancras Parish. R. B. Smith, L.R.C.P.Edin., L.R.C.S.I., appointed Medical Officer for the Burton Hastings District of the Nuneaton Union. J. J. Spears, appointed Medical Officer for the Abrewas District of the Lichfield Union. A. Tuxford, M.D., appointed Medical Officer of Health to the Urban Sanitary Authority, Boston, for three years, and to the Sibsey Rural District Council until April, 1899. J. P. Wagstaff, L.R.C.P., M.R.C.S., appointed Medical Officer for the No. 4 District of the Saffron Walden Union. F. Warner, M.D.Lond., F.R.C.P., F.R.C.S., appointed Full Physician to the London Hospital.

**PASS LIST.**

**University of Durham.**

First Examination for the Degree of Bachelor in Medicine.

The following candidates have satisfied the Examiners :—

Chemistry with Chemical Physics, and Botany with Medical Botany.—Old regulations: E. G. Klumpp, St. Bartholomew's Hospital.

Elementary Anatomy and Biology, Chemistry and Physics.—Honours, First Class: R. Alderson, College of Medicine, Newcastle-upon-Tyne. Honours, Second Class: J. R. Burn, College of Medicine, Newcastle-upon-Tyne; F. W. Lambelle, College of Medicine, Newcastle-upon-Tyne; D. M. Johnston, College of Medicine, Newcastle-upon-Tyne; E. T. Born, College of Medicine, Newcastle-upon-Tyne. Pass List: J. W. H. Morrison, College of Medicine, Newcastle-upon-Tyne; H. R. Moxon, College of Medicine, Newcastle-upon-Tyne; T. S. P. Parkinson, College of Medicine, Newcastle-upon-Tyne; R. Peart, College of Medicine, Newcastle-upon-Tyne; T. Russell, College of Medicine, Newcastle-upon-Tyne; Eleanor Shepheard, London School of Medicine for Women.

Chemistry and Physics.—Hedda Alström, London School of Medicine for Women; C. O. Bodman, University College, Bristol; J. J. French, College of Medicine, Newcastle-upon-Tyne; H. G. Harris, St. Bartholomew's Hospital; H. W. Horan, College of Medicine, Newcastle-upon-Tyne; Margaret Joyce, London School of Medicine for Women; J. R. Mitchell, College of Medicine, Newcastle-upon-Tyne; H. R. D. Spitta, St. George's Hospital; Grace Harwood Stewart, London School of Medicine for Women; A. M. Thomas, Guy's Hospital; H. Widdas, College of Medicine, Newcastle-upon-Tyne.

Anatomy and Biology.—S. T. Cochrane, College of Medicine, Newcastle-upon-Tyne; F. Clarkson, College of Medicine, Newcastle-upon-Tyne; S. J. S. Cooke, College of Medicine, Newcastle-upon-Tyne; C. H. Gibson, College of Medicine, Newcastle-upon-Tyne; E. J. L. Kendle, B.A., College of Medicine, Newcastle-upon-Tyne; R. T. Vaux, College of Medicine, Newcastle-upon-Tyne; S. Raw, College of Medicine, Newcastle-upon-Tyne; E. E. P. Taylor, College of Medicine, Newcastle-upon-Tyne.

Elementary Anatomy, Chemistry, and Physics.—W. Asten, Mason College, Birmingham; H. Braund, Guy's Hospital; R. T. Jupp, Mason College, Birmingham; P. M. Perkins, St. Bartholomew's Hospital; R. Thorne-Thorne, jun., St. Bartholomew's Hospital; H. B. Thompson, Mason College, Birmingham; W. Wakefield, Mason College, Birmingham.

Elementary Anatomy and Chemistry.—J. H. Stormont, Mason College, Birmingham.

